# The Requirement and Protective Effects of Dietary Protein against Chronic Ammonia Exposure in Juvenile Genetically Improved Farmed Tilapia (*Oreochromis niloticus*)

**DOI:** 10.1155/2023/6306517

**Published:** 2023-05-30

**Authors:** Xing Lu, Zhe-Hui Ji, Mei-Zi Wang, Juan Tian, Li-Xue Dong, Zhong-Bao Guo, Yong-Ju Luo, Hua Wen, Ming Jiang

**Affiliations:** ^1^Yangtze River Fisheries Research Institute, Chinese Academy of Fishery Sciences, Wuhan 430223, China; ^2^Guangxi Institute of Fisheries, Nanning 530221, China

## Abstract

Ammonia is a key risk factor in intensive aquaculture systems. This experiment is aimed at investigating the influence of dietary protein levels on genetically improved farmed tilapia (GIFT, *Oreochromis niloticus*) under chronic ammonia stress. GIFT juveniles of 4.00 ± 0.55 g were exposed to high ammonia level at 0.88 mg/L and fed with six diets comprising graded protein levels at 22.64%, 27.26%, 31.04%, 35.63%, 38.47%, and 42.66% for 8 weeks. The fish in negative control was fed the diet with 31.04% protein in normal water (0.02 mg ammonia/L water). Our results showed that high ammonia exposure (0.88 mg/L) caused significant decrease in fish growth performance, hematological parameters, liver antioxidant enzymes (catalase and glutathione peroxidase), and gill Na^+^- and K^+^-dependent adenosine triphosphatase (Na^+^/K^+^-ATP) activity. When fish were under high ammonia exposure, the weight gain rate, special growth rate, feed efficiency, and survival rate elevated significantly with dietary protein supplementation increase to 35.63%, whereas protein efficiency ratio, hepatosomatic index, and viscerosomatic index showed a decreased tendency. Dietary protein administration significantly enhanced crude protein but reduced crude lipid contents in the whole fish. Fish fed diets with 35.63%-42.66% protein had higher red blood cell counts and hematocrit percentage than fish fed 22.64% protein diet. The values of serum biochemical indices (lactate dehydrogenase, aspartate aminotransferase, and alanine aminotransferase), hepatic antioxidant enzymes (superoxide dismutase, catalase, and glutathione peroxidase), and gill Na^+^/K^+^-ATP activity were all elevated with the increment of dietary protein. Moreover, histological analysis indicated that dietary protein administration could prevent the ammonia-induced damages in fish gill, kidney, and liver tissues. Based on weight gain rate as a response criterion, the optimal dietary protein requirement for GIFT juveniles under chronic ammonia stress was 37.9%.

## 1. Introduction

In aquaculture systems, high stocking densities could easily generate long-term ammonia stress by reason of the ammonification of feed residual and animal excreta. Ammonia is reported to present in aquatic environments as either unionized (NH_3_) or ionized (NH_4_^+^) form, and most of the teleost are extremely sensitive to toxic NH_3_ because the gas can pass across fish gills with its lipid solubility and noncharge property [1]. High ammonia levels, mainly produced from intensive factory farming, have detrimental effects on feeding behaviors and health of the cultured species such as growth retardation, blood chemistry disorders, oxidative damage, tissue degeneration, immune suppression, and even individual death [2–5]. Therefore, excessive NH_3_ has been regarded as an alarming risk factor that poses a serious threat to sustainable aquaculture development.

Dietary manipulations are good means to alleviate the ammonia poisoning on aquatic animals. For instance, Li et al. [6] showed that increasing dietary linolenic acid levels from 459.7 to 993.8 mg/kg can enhance *Pelteobagrus vachelli* resistance to acute ammonia exposure (5.70 mg/L total NH_3_-N) in terms of the requirement of essential fatty acids. Hoseini et al. [7] indicated that supplement of 0.5% myrcene and 0.25% menthol, derived from herbal products with antioxidant and anti-inflammatory efficacy, assists *Cyprinus carpio* to reduce the tissue damage and anemia triggered by acute ammonia stress (0.5 mg/L). Zhang et al. [8] found that exogenous Se addition that ranged from 0.30 to 0.60 mg/kg can attenuate the adverse effects of ammonia toxicity (5.70-11.40 mg/L) on growth, oxidative damage, immunosuppression, and apoptosis of *Pelteobagrus fulvidraco*. Furthermore, Chen et al. [9] reported that *Litopenaeus vannamei* fed with higher levels of inositol from 459.7 to 993.8 mg/kg effectively strengthen tolerance to acute ammonia treatment (60.21 mg/L) by suppressing fat peroxidation and protein oxidation degrees and elevating antioxidant enzyme activities.

Proteins are considered as the most expensive and indispensable nutrients that provide the essential and nonessential amino acids for fish growth and metabolism. They are involved in a myriad of physiological events as functional enzymes and thus potentiate to boost body growth, antioxidant status, and stress resistance [10]; however, excess protein in diets would be metabolized for energy use, leading to augment ammonia nitrogen excretion and may deteriorate the quality of aquaculture water [11, 12]. Both insufficient and excessive protein diets not only affect fish growth and health but also influence water quality as well as total expenditure in fish cultivation; it is thereby desirable to employ the minimal protein sources that provide necessary amino acids for fish to maintain the best growth performance and development. Hence, the optimization of dietary protein requirement in aquaculture is imperative in consideration of the economic cost and friend farming environment.

Tilapia (*Oreochromis niloticus*) is an important commercial fish because of its advantages of rapid growth, disease resistance, fillet production, and high market shares [13]. The genetically improved farmed tilapia (GIFT) strain of Nile tilapia was developed by the good merits of superior growth rates, better flesh quality, and increased resistance to emerging diseases in aquaculture [14, 15]. GIFT has become one of the most popular species that is widely cultured in about 87 countries [16]. It is reported that the dietary protein level for tilapia juveniles was approximately 30% under normal rearing conditions [17]. As production demands and reliance on high-density aquaculture industry heighten, the elevated concentrations of ammonia will subject this species to issues associated with chronic ammonia toxicity. Consequently, the intensification of tilapia breeding requires evaluating the nutrient requirement and its benefits against ammonia stress.

Herein, this study was conducted to explore the dietary protein supplementation on tilapia growth performance, blood status, antioxidant-related enzyme activities, and tissue structure in resistance to chronic ammonia challenge. These data would illustrate dietary protein requirement under chronic ammonia exposure and the protective effects of dietary protein against chronic ammonia toxicity in tilapia.

## 2. Material and Methods

### 2.1. Ethical Clearance Statement

This research was approved by the committee of Animal Ethics of Yangtze River Fisheries Research Institute, Chinese Academy of Fishery Sciences. Fish management and sampling were in accordance with the guidance of animal care procedures.

### 2.2. Diets

Six isoenergetic diets were prepared to include different concentrations of crude protein at 22.64%, 27.26%, 31.04%, 35.63%, 38.47%, and 42.66%, respectively. Casein and gelatin were utilized as protein ingredients, coin oil and soybean oil as lipid ingredients, and dextrin as carbohydrate ingredients. Diet formulation and proximate analysis are displayed in Table 1. The procedures of diet preparation were followed as our published literature [17].

### 2.3. Feeding Management

GIFT juveniles were transported from the Guangxi Tilapia National Breeding Farm (Nanning, China). Fish were fed with the commercial diet in an indoor recirculating aquarium system (RAS) at Yangtze River Fisheries Research Institute (Wuhan, China). The RAS included 18 polycarbonate tanks (500 L each, diameter = 100 cm, water depth = 73.5 cm), which was provided with recirculating water flow (about 5 L/min) and continuous aeration to maintain the dissolved oxygen (DO) concentrations. After 2-week adaptation, the healthy and uniform-sized tilapia juveniles were selected for subsequent feeding trial.

In this study, high ammonia level was set at 0.88 mg/L based on half dose of 96 h-LC_50_ (1.76 mg/L); the value was determined in a preacute toxicity test. Ammonia chloride (NH_4_Cl) solution at 10 g/L was made and then diluted to the desired dosage with tank water. The actual ammonia-nitrogen content in water was measured every 24 h by nesslerization and then converted to unionized ammonia concentration according to the equation reported previously [6]. A negative control was also set up; the fish in that were reared in another independent culture system like the one mentioned above and fed the diet with 31.04% protein in normal water (0.02 mg ammonia/L water). After 24 h starvation, fish with an initial weight of 4.00 ± 0.55 g were randomly distributed into 7 groups. Each group was composed of 3 replicated tanks with 40 individuals. Fish in one group were fed with 31.04% protein diet under 0.02 mg/L ammonia level, while fish in the remaining six groups were fed with above six experimental diets under 0.88 mg/L ammonia level. Each diet was at random allocated to fish in three tanks by hand to apparent satiety within half hour thrice a day (8:00, 12:00, and 16:00). The feed intake in every tank was monitored. To maintain ammonia concentrations, the half water in chronic ammonia exposure groups was replaced with fresh water while ammonium chloride was replenished and pH was adjusted every 2 days. During the feeding, water temperature was kept at 28 ± 0.5°C, pH was 7.0 ± 0.5°C, and DO > 5.0 mg/L. The feeding trial was conducted at a natural photoperiod and lasted for 8 weeks.

### 2.4. Sample Collection

At the end of feeding trial, all fish were deprived of feeds for 24 h. Then, fish were batch-weighed and counted to calculate fish weight and total survival. Six tilapias were randomly caught from each tank and then anaesthetized with 80 mg/L MS-222 (Sigma-Aldrich, USA). Among them, three individuals were used for measuring the condition factor (CF) and stored at -20°C for later whole-fish composition analysis. Blood was collected from another three individuals by puncturing fish caudal vein, coagulated at 4°C, and centrifuged at 1000*g* to separate serum. The blood and serum samples were cryopreserved at -80°C for the following hematological assessment and serum biochemical determination. Subsequently, these three fish were killed on ice to isolate the target tissues (including gill, liver, viscus, and kidney), and data of hepatosomatic index (HSI) and viscerosomatic index (VSI) were obtained subsequently. A portion of gill, liver, and kidney tissues was immediately fixed in 4% formaldehyde for the histological measurement. Another part of livers was pooled and snap-frozen for the enzyme activity examination.

### 2.5. Sample Analysis

Experimental feeds and whole fish were conducted for proximate composition according to standard methods of the Association of Official Agricultural Chemists [18]. Briefly, crude protein was determined by the Kjeldahl method; crude lipid was examined by Soxhlet extraction; moisture was detected by the freeze-drying method; ash was assessed through combustion in a muffle furnace at 550°C; gross energy was measured using an isothermal automatic calorimeter (SDACM 4000).

The blood parameters, including red blood cell (RBC) counts and hematocrit (Ht) percentage, were measured within 6 hours using a full-automatic veterinary blood cell analyzer (BC-2800vet).

Serum biochemical indices including content of total protein (TP) as well as activities of lactic dehydrogenase (LD), aspartate aminotransferase (AST), and alanine aminotransferase (ALT) were analyzed by an automatic blood analyzer (CHEMIX-800, Mikan Hisen, Japan) with commercial assay kits (Sysmex Wuxi Co., Ltd., Wuxi, China).

Antioxidant enzyme parameters in liver extracts including activities of superoxide dismutase (SOD), catalase (CAT), and glutathione peroxidase (GPx) were determined as described previously [19] with commercial kits from Nanjing Jiancheng Bioengineering Institute, China. The activity of Na^+^/K^+^-ATP in gill samples was measured kinetically using a commercial kit following the manufacturers' instruction (Nanjing Jiancheng Bioengineering Institute, China).

Fixed tissues including gill, liver, and kidney for histology were performed as reported previously [20]. Section series of 5 *μ*m were processed with hematoxylin and eosin (H&E) and scanned using an Olympus CX41 microscope (Olympus, Tokyo, Japan).

### 2.6. Statistical Analysis

Results were displayed as mean ± SD (standard deviation) of three replicates. All data were subjected to one-way analysis of variance (ANOVA). When differences were measured to be significant at *P* < 0.05, Tukey's test was utilized to determine mean values among treatment groups. Statistical analysis was undertaken using SPSS 19.0 for Windows (Chicago, USA). The broken-line model [21] analysis was used to evaluate the optimal dietary protein requirement for GIFT juveniles.

## 3. Results

### 3.1. Growth Performance and Physical Indexes

**Figure 1:**
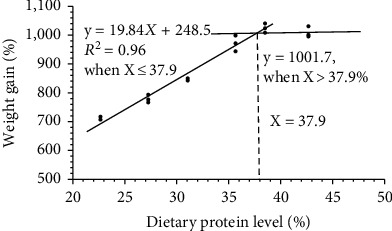
Broken-line regression analysis of the relationship between dietary protein levels and weight gain rate of GIFT juveniles.

As shown in Table 2, fish reared at high ammonia level (0.88 mg/L) for 8 weeks had a decrease of final body weight (FBW), weight gain rate (WGR), specific growth rate (SGR), feed efficiency (FE), and survival rate (SR), whereas there is increase of protein efficiency ratio (PER) compared to those at low ammonia level (0.02 mg/L). When fish were chronically exposed to 0.88 mg/L ammonia, the FBW, WGR, SGR, FE, and SR increased remarkably with increasing dietary protein supplementation from 22.64% to 35.63%, but afterwards kept almost unchanged with further raising in dietary protein. Fish fed 22.64% protein diet had the lowest WGR, SGR, FE, and SR. However, PER declined significantly as dietary protein level increase to 42.66% (*P* < 0.05). Considering WGR as response criteria, the broken-line regression model indicated that the optimal dietary protein level for GIFT juveniles under high ammonia exposure was 37.9% (Figure 1).

Physical indexes of GIFT are shown in Table 3. The condition factor (CF) of fish under low ammonia was significantly higher than that under high ammonia stress (*P* < 0.05). The values of hepatosomatic index (HSI) and viscerosomatic index (VSI) in fish exposure to 0.88 mg/L ammonia for 8 weeks were decreased markedly as protein level increased from 22.64% to 38.47%.

### 3.2. Whole-Body Proximate Composition

The whole-body proximate compositions are shown in Table 4. When fish were subjected to 0.88 mg/L ammonia, the contents of moisture and crude protein raised continuously as elevating protein concentrations from 22.64% to 42.66%, while crude lipid content exhibited a decreased tendency as increasing protein level up to 38.47%. The maximum values of crude protein and minimum crude lipid were observed in the 42.66% protein and 38.47% protein groups, respectively. However, ash content had no significant difference among all dietary groups (*P* > 0.05).

### 3.3. Hematology and Serum Biochemical Indices

High ammonia treatment resulted in significant reduction in red blood cell (RBC) counts and hematocrit (Ht) percentage (*P* < 0.05) (Table 5). When fish were under 0.88 mg/L ammonia stress, the RBC value in 35.63%-42.66% protein groups was significantly higher than that in 22.64%-31.04% protein groups (*P* < 0.05), except for the 27.26% protein group. Fish fed diets with 35.63%-42.66% protein had greater Ht than fish fed diet containing 22.64% protein irrespective of ammonia level (Table 5).

The results for serum biochemical indices are listed in Table 5. When fish were exposed to 0.88 mg/L ammonia, increasing dietary protein levels significantly enhanced activities of lactate dehydrogenase (LD), aspartate aminotransferase (AST), and alanine aminotransferase (ALT) (*P* < 0.05). Their values were the highest in the 42.66% protein group compared to other groups. The total protein (TP) content in the 35.63% protein group was significantly higher than that in the 22.64% protein group (*P* < 0.05).

### 3.4. Liver and Gill Enzyme Activities

The data on liver antioxidant enzymes including superoxide dismutase (SOD), catalase (CAT), glutathione peroxidase (GPx), and gill Na^+^/K^+^-ATP are shown in Table 6. In confrontation to exposure to low ammonia level, high ammonia stress caused significant decrease in CAT and GPx activities in fish fed the diet with 31.04% protein. When fish were exposed to 0.88 mg/L ammonia, the SOD, CAT, GPx, and Na^+^/K^+^-ATP amounts increased obviously as elevating protein levels in diets. And the highest values of them were obtained in the 42.66% protein group compared to the 22.64% protein group (*P* < 0.05).

### 3.5. Tissue Morphology

**Figure 2:**
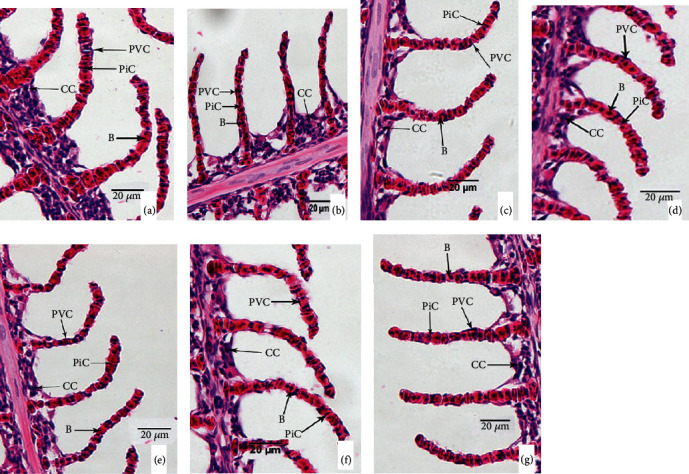
Gill histologic alterations of GIFT juveniles fed diets with different protein levels under low or high ammonia exposure for 8 weeks (×400; scale bar = 20 *μ*m; CC: chloride-secreting cell; PVC: pavement cell; Pic: pillar cell; B: blood cell). (a) The fish were fed diet with 31.04% protein under 0.02 mg/L ammonia exposure. (b)–(g) The fish were fed diets with 22.64%, 27.26%, 31.04%, 35.63%, 38.47%, and 42.66% protein under 0.88 mg/L ammonia exposure, respectively.

**Figure 3:**
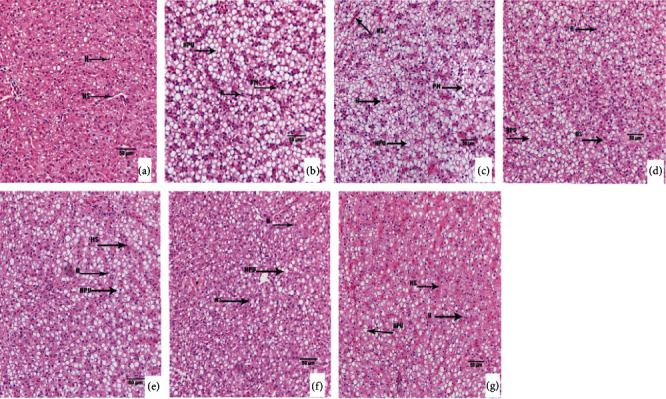
Liver histologic changes of GIFT juveniles fed diets with different protein levels under low or high ammonia exposure for 8 weeks (×400; scale bar = 50 *μ*m; H: hepatocyte; HS: hepatic sinusoid; NM (PN): nuclear migration; HPV: hepatic vacuolization). (a) The fish were fed diet with 31.04% protein under 0.02 mg/L ammonia exposure. (b)–(g) The fish were fed diets with 22.64%, 27.26%, 31.04%, 35.63%, 38.47%, and 42.66% protein under 0.88 mg/L ammonia exposure, respectively.

**Figure 4:**
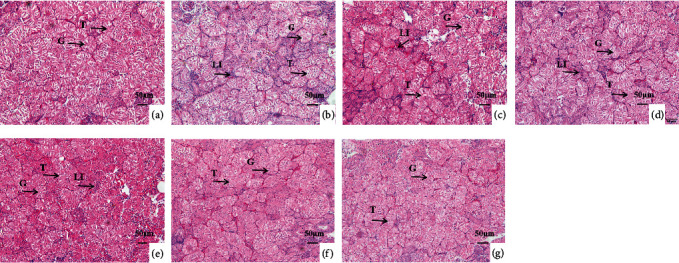
Kidney histologic alterations of GIFT juveniles fed diets with different protein levels under low or high ammonia exposure for 8 weeks (×400; scale bar = 50 *μ*m; T: renal tubule; G: glomerulus; LI: lymphocyte infiltration). (a) The fish were fed diet with 31.04% protein under 0.02 mg/L ammonia exposure. (b)–(g) The fish were fed diets with 22.64%, 27.26%, 31.04%, 35.63%, 38.47%, and 42.66% protein under 0.88 mg/L ammonia exposure, respectively.

Effects of ammonia exposure on histological alteration in tilapia gill, kidney, and liver tissues were investigated. For gills, high ammonia exposure caused irregular arrangement of pillar cells (PiC), exfoliation of pavement cells (PVC), and vacuolization of chlorine-secreting cells (CC) (Figures 2(a)–2(d)). For livers, high ammonia stress led to serious vacuolization and nuclear migration of hepatocytes (Figures 3(a)–3(d)). For kidneys, high ammonia treatment resulted in lymphocyte infiltration (Figures 4(a)–4(d)). However, dietary protein administration could prevent the ammonia-induced tissue damages. Compared to those fed lower protein diets (22.64%-31.04%), fish fed higher protein diets (35.63%-42.66%) showed the lighter or normal cell morphology in gills (Figures 2(e)–2(g)), livers (Figures 3(e)–3(g)), and kidneys (Figures 4(e)–4(g)). The histopathology evaluation of gill and kidney tissues of GIFT juveniles fed diets with different protein levels under low or high ammonia exposure is listed in Table 7.

## 4. Discussion

Ammonia is an important parameter that easily accumulates in intensive aquaculture, while high levels of unionized ammonia can pose a threat to fish survival. In this study, the 96 h LC_50_ data for acute ammonia toxicity on GIFT juveniles was determined at 1.76 mg/L, which is higher than that on *Centropristis striata* at 0.46-0.54 mg/L [22] and *Lota lota* at 0.58 mg/L [23]. The differences in sensitivity to ammonia stress could be dependent on fish species, individual size, and various ammonia detoxification abilities. Besides, high ammonia exposure also resulted in the retardation of fish growth, and feed efficiency (FE) was closely correlated with the concentrations of ammonia exposure [24]. In the present study, final body weight (FBW), weight gain rate (WGR), and specific growth rate (SGR) of GIFT juveniles treated with high ammonia level were significantly lower than those under low ammonia due to the low value of feed efficiency. Several parallel findings are published in other fish species, such as *Scophthalmus maximus* [25], *Megalobrama amblycephala* [26], and *Pelteobagrus vachelli* [6]. When fish were under chronic ammonia stress, it may need more energy substrates from feed to eliminate the adverse effects [27]. The results from this study revealed that dietary protein levels significantly improved the fish growth during the 8-week exposure experiment, and the optimal dietary protein content for GIFT juveniles to reach maximum WGR was 37.9%. A similar study in our lab on protein requirements in tilapia under chronic cold stress showed that tilapia increased their protein requirements [17]. These two experiments suggest that tilapia may need more protein to maintain growth and health under stress. Although the growth performance of GIFT was ameliorated by increasing dietary protein, the growth performance of tilapia under stress condition could only reach about 70% of that under normal culture condition. This study and previous study [17] confirmed that the aquaculture environmental control is the key measure to maintain the normal growth of tilapia.

The values of hepatosomatic index (HSI), viscerosomatic index (VSI), and condition factor (CF) are usually used for monitoring the energy status in fish. In our study, HSI and VSI declined linearly as elevating dietary protein supplementation from 22.64% to 38.47%. This may be associated with high carbohydrate concentrations in low protein diets that induced the glycogen deposition or fat accumulation in the liver [28]. Regardless of dietary protein levels, chronic ammonia exposure seemingly lowered the CF in fish, which may be triggered by the decrease of feed efficiency and consequently resulted in the reduction of body weight. It is reported that supplement with different protein levels could influence whole-body compositions in fish [17]. In the current study, the contents of crude protein and moisture increased continuously, whereas the crude lipid decreased as the rise of dietary protein. Similar findings have been evident on *Diplodus puntazzo* [29] and *Epinephelus akaara* [30]. Nevertheless, other researches indicated that the crude protein content was in indirect proportion to dietary protein levels, which might be related to feed formulation or amino acid composition in diets. Besides, ammonia stress had no significant influence on crude lipid content in fish body, which is inconsistent with Li et al. [24] who reported that high ammonia caused a decrease in whole-body lipid as a result of the utilization of energy storage. Thus, the impacts of chronic ammonia exposure on fish whole-body compositions need to be further studied.

Hematological parameters have been deemed as sensitive indicators to determine the fish health in response to ammonia stress or dietary manipulations [31]. In the present experiment, red blood cell (RBC) counts and hematocrit (Ht) percentage of GIFT juveniles reared at high ammonia were significantly lower than those at low ammonia. These results are in accordance with other reports by Hoseini et al. [7] and Li et al. [24], which indicate that ammonia is toxic to fish by reducing red blood cells and hematocrit. On the other side, dietary protein levels could enhance the hematological parameters in several fish species such as *Megalobrama amblycephala* [32] and *Oncorhynchus mykiss* [33]. Our data also demonstrated a significant increase in RBC and Ht values with the increment of dietary protein supplementation from 35.63% to 42.66%, suggesting that higher protein diets contribute to the intake of oxygen and thereby improve the fish survival during the stressful conditions. Serum biochemical indices including lactate dehydrogenase (LD), aspartate aminotransferase (AST), and alanine aminotransferase (ALT) are used to reflect the physiological changes of fish following environmental stress [31]. LD is reported to be involved in anaerobic metabolism which catalyzes the conversion of lactate to pyruvate [34]. AST and ALT are known as key enzymes for assessing the damage to liver function [19]. In this study, regardless of ammonia exposure, dietary protein levels significantly enhanced activities of serum LD, AST, and ALT in GIFT juveniles, indicating that tilapia have the capability of accommodating protein catabolism to protein input. Previous literatures have also reported that AST and ALT activities had a positive correlation to the increment of dietary protein level [12, 35]. The content of TP in serum significantly elevated as dietary protein concentration from 22.64% to 35.63%, which might be ascribed to the accumulation of digested protein in fish body and provide more energetic demands in response to ammonia stress.

Fish are reported to possess antioxidant defense ability to protect cells against oxidative stimulation by the generation of reactive oxygen species (ROS). Several representative antioxidant enzymes (including SOD, CAT, and GPx) widely exist in tissues with high abundance in the liver; they can reduce the ROS accumulation via catalyzing hydrogen peroxide into water and oxygen. In this study, elevating dietary protein level from 22.64% to 42.66% significantly improved the activities of SOD, CAT, and GPx in tilapia liver, suggesting that the protective effects of dietary protein may be associated with the increase in antioxidant enzyme activities. This finding is consistent with the conclusion of Xu et al. [36] that appropriate increases in dietary protein content can improve fish resistance to a certain extent. The Na^+^/K^+^-ATPase is a protease from the membrane of gill chloride-secreting cells and organelles, which exerts crucial functions in fish osmoregulation [37]. It is shown that a negative correlation between higher ammonia nitrogen concentrations and Na^+^/K^+^-ATPase activity was found in *Macrobrachium nipponense* [38]. In this study, the Na^+^/K^+^-ATPase activity in tilapia gill exposed to high ammonia level was lower compared to that exposed to low ammonia, indicating that chronic ammonia stress affects the protein structure of the Na^+^/K^+^-ATPase, leading to a reduction in enzyme activity and dysfunction of osmoregulation. The decrease in Na^+^/K^+^-ATPase may be related to ammonia stress leading to the detachment of gill respiratory epithelial cells and hyperemia of gills. However, a significant enhancement in Na^+^/K^+^-ATPase activity was detected when fish were fed the 31.04%-42.66% protein diet, indicating that appropriate dietary protein levels could attenuate the adverse effects of hyperammonia on tilapia.

The fish gill is an important respiratory organ with the functions of respiration, filtration, ammonia-N excretion, and osmoregulation. Lease et al. [39] reported that fish gill is in direct contact with the environmental stress or contamination, which may easily cause the damage of gill physiological function and tissue structure. Moreover, stress also results in the hyperplasia of epithelial cells, edema of lamellae respiratory epithelial cells, and even necrosis and abscission in gills [31]. Nonionic ammonia is fat-soluble and can penetrate and diffuse freely across the gill membrane, which led to cell damage in gill tissues. Long-term ammonia stress disrupts the balance of defense systems, resulting in damage to fish gill tissue cells, impaired respiration, and fish mortality. Here, the results from our experiment showed that high ammonia level had significant effects on the gill structure of GIFT juveniles. Ammonia stress caused the gill respiratory epithelial cells to be partially detached, pillar cells (Pic) to be irregularly arranged, and chloride-secreting cells (CC) to be not only widely vacuolated but also reduced a lot in number in tilapia gills. Benli et al. [2] found that Nile tilapia showed dilated capillaries, increased numbers of chloride-secreting cells, clustering of gill lamellae, and congestion under chronic ammonia stress. In this experiment, the gills of GIFT juveniles showed a large number of vacuolation of chloride-secreting cells under ammonia stress, which is in keeping with the results of Zhang et al. [26] who indicated that cytoplasmic vacuolation and epithelial cell detachment in gills were observed in *Megalobrama amblycephala* exposed to different ammonia levels. As the protein level increased, the vacuolation of chlorine-secreting cells gradually decreased, the pillar cell arrangement became regular, and the dropping of respiratory epithelial cells on the gill lamellae was reduced. To a certain extent, increasing the protein level in the feed can reduce the damage to tilapia gill tissue caused by ammonia stress.

The liver is a primary detoxification and metabolic organ in which the clinical symptom firstly occur under environmental stress. Previous studies have revealed that high-level ammonia treatment could induce the liver structural damage in fish with swelling, vacuolization, and localized necrosis [31]. Our results of the present study displayed that high concentrations of ammonia stress had significant influences on the liver structure in GIFT juveniles, causing partial vacuolization of the hepatocytes and nuclear migration. Similar results were found in other pollutants, such as Mishra and Mohanty [40] pointed out that the hepatocytes of *Channa punctata* showed vacuolization under heavy metal pollution. Without consideration of ammonia concentration, a decreasing trend of vacuolation in tilapia hepatocytes was observed as dietary protein level increased, which indicate that dietary protein administration, to a certain extent, can reduce the liver destruction in ammonia-stressed tilapia. The kidney is an excretory organ in fish and can excrete the metabolic products from the body. Ammonia exposure also resulted in severe histological alterations in the vacuolation of renal tubules, lymphocyte infiltration, and glomerulonephritis. Results of this research showed that high concentration of ammonia stress caused the lymphocyte infiltration in tilapia kidney, while increasing dietary protein levels are beneficial for alleviating the kidney structural damage. These data indicated that chronic ammonia exposure is harmful to tilapia tissues, and dietary protein administration could reduce the ammonia-induced adverse effects.

## 5. Conclusion

The present study demonstrated that chronic ammonia exposure caused significant decrease in fish growth performance, hematological parameters, and liver antioxidant enzymes (catalase and glutathione peroxidase), as well as gill Na^+^/K^+^-ATP activity. Under 0.88 mg/L ammonia exposure, fish growth, feed efficiency, and survival rate increased significantly with dietary protein level rise to 35.63%. Dietary protein administration significantly enhanced whole-body crude protein content, red blood cell counts and hematocrit percentage, serum biochemical indices (lactate dehydrogenase, aspartate transaminase, and alkaline phosphatase), liver antioxidant enzymes (superoxide dismutase, catalase, and glutathione peroxidase), and gill Na^+^/K^+^-ATP activity. Moreover, histological analysis indicated that dietary protein administration could prevent the ammonia-induced damages in fish gill, kidney, and liver tissues. Based on weight gain rate as a response criterion, the optimal dietary protein requirement for GIFT juveniles under chronic ammonia stress was 37.9%.

## Figures and Tables

**Table 1:** Ingredients and proximate compositions of the experimental diets (% air dry matter).

	Dietary protein level (%)					
	22.64	27.26	31.04	35.63	38.47	42.66
Feed ingredients (%)						
^a^Casein	21.60	25.60	29.60	33.60	37.60	41.60
^a^Gelatin	5.40	6.40	7.40	8.40	9.40	10.40
^a^Dextrin	48.00	42.00	36.00	30.00	24.00	18.00
^b^Corn oil	3.45	3.45	3.45	3.45	3.45	3.45
^b^Soybean oil	3.45	3.45	3.45	3.45	3.45	3.45
^c^Vitamin premix	1.00	1.00	1.00	1.00	1.00	1.00
^d^Mineral premix	2.00	2.00	2.00	2.00	2.00	2.00
^e^Choline chloride	0.25	0.25	0.25	0.25	0.25	0.25
^e^Ca(H_2_PO_4_)_2_	2.00	2.00	2.00	2.00	2.00	2.00
^e^Bentonite	2.00	2.00	2.00	2.00	2.00	2.00
^e^Microcellulose	10.85	11.85	12.85	13.85	14.85	15.85
Total (%)	100	100	100	100	100	100
Proximate composition (%)						
Dry matter	96.01	95.95	95.35	95.39	95.30	96.11
Crude protein	22.64	27.26	31.04	35.63	38.47	42.66
Crude lipid	6.79	7.08	7.03	7.19	6.99	7.19
Ash	5.05	5.13	5.14	5.09	5.10	5.35
Gross energy (KJ/g)	15.52	15.79	15.73	15.94	15.59	15.73

^a^Wuhan Xilong Chemical Co. ^b^Wuhan Coland Co. ^c^Vitamin mixture (g/kg): thiamine hydrochloride, 5; riboflavin, 5; calcium pantothenate, 10; nicotinic acid, 6.05; biotin, 0.03; pyridoxine, 4; folic acid, 1.5; inositol, 200; L-ascorbyl-2-polyphosphate, 3.95; tocopherol, 5; menadione, 4; retinol, 0.4; cholecalciferol, 18.74 IU. All ingredients were diluted with microcellulose to 1 g. ^d^Mineral mixture (g/kg): C_6_H_10_CaO_6_, 500; FeSO_4_·7H_2_O, 20; MgSO_4_, 100; NaH_2_PO_4_, 100; NaCl, 20; AlCl_3_, 0.6; KIO_3_, 0.6; KCl, 40; CuSO_4_, 2; MnSO_4_, 4; CoCl_2_, 2; ZnSO_4_, 20. All ingredients were diluted with microcellulose to 1 g. ^e^COFCo.

**Table 2:** Growth performance of GIFT juveniles fed diets with different protein levels under low or high ammonia exposure for 8 weeks.

Ammonia (mg/L)	Protein level (%)	IBW	FBW	WGR	SGR	FE	PER	SR
0.02 mg/L	31.04	4.27 ± 0.19	68.52 ± 1.65^d^	1480.55 ± 21.69^d^	4.95 ± 0.15^d^	0.81 ± 0.01^a^	2.65 ± 0.10^c^	95.83 ± 2.01^b^
0.88 mg/L	22.64	4.43 ± 0.04	34.37 ± 3.10^a^	712.46 ± 6.03^a^	3.74 ± 0.01^a^	0.92 ± 0.01^b^	4.73 ± 0.11^g^	85.00 ± 1.73^a^
	27.26	4.41 ± 0.02	38.22 ± 3.02^ab^	780.34 ± 18.21^ab^	3.78 ± 0.11^a^	0.94 ± 0.02^b^	3.75 ± 0.07^f^	90.83 ± 1.44^ab^
	31.04	4.41 ± 0.13	42.04 ± 1.83^bc^	842.70 ± 0.70^b^	4.03 ± 0.03^b^	0.98 ± 0.01^bc^	3.27 ± 0.04^e^	93.33 ± 5.20^b^
	35.63	4.57 ± 0.06	49.27 ± 1.05^c^	971.52 ± 27.90^c^	4.23 ± 0.06^c^	1.04 ± 0.02^cd^	2.90 ± 0.09^d^	95.00 ± 2.50^b^
	38.47	4.42 ± 0.13	48.90 ± 0.98^c^	1024.14 ± 16.09^c^	4.32 ± 0.04^c^	1.08 ± 0.03^d^	2.68 ± 0.05^c^	94.50 ± 3.46^b^
	42.66	4.52 ± 0.09	45.75 ± 3.64^bc^	1013.21 ± 24.32^c^	4.15 ± 0.16^bc^	1.00 ± 0.01^c^	2.49 ± 0.01^b^	94.16 ± 1.44^b^

^∗^Values are mean ± SD (standard deviation) of three replicate per treatment, values in the same row within the same protein level sharing different letters are significantly different at *P* < 0.05, as determined by Tukey's test. IBW (g/fish) = initial body weight; FBW (g/fish) = final body weight; WGR (weight gain rate, %) = 100 × (FBW − IBW)/IBW; SGR (specific growth rate, %/day) = 100 × (ln FBW − ln IBW)/feeding days; FE (feed efficiency) = (g total final fish weight − g total initial fish weight + g dead fish)/(g fish intake); PER (protein efficiency ratio) = 100 × (g weight gain)/(g protein intake); SR (survival rate, %) = 100 × final fish number/initial number.

**Table 3:** Physical indexes of GIFT juveniles fed diets with different protein levels under low or high ammonia exposure for 8 weeks.

Ammonia (mg/L)	Protein level (%)	HSI (%)	VSI (%)	CF (%)
0.02 mg/L	31.04	1.21 ± 0.04^ab^	9.32 ± 0.18^cd^	3.54 ± 0.16^ab^
0.88 mg/L	22.64	2.33 ± 0.02^d^	9.99 ± 0.40^d^	3.33 ± 0.08^a^
	27.26	2.22 ± 0.36^cd^	8.87 ± 0.52^cd^	3.48 ± 0.04^a^
	31.04	1.78 ± 0.09^bcd^	8.08 ± 0.32^abc^	3.60 ± 0.09^ab^
	35.63	1.68 ± 0.11^bc^	7.49 ± 0.68^ab^	3.66 ± 0.05^b^
	38.47	1.39 ± 0.15^b^	6.81 ± 0.41^a^	3.53 ± 0.16^ab^
	42.66	1.59 ± 0.26^b^	7.06 ± 0.34^a^	3.51 ± 0.13^ab^

^∗^Values are mean ± SD (standard deviation) of three replicate per treatment, values in the same row within the same protein level sharing different letters are significantly different at *P* < 0.05, as determined by Tukey's test. HSI (hepatosomatic index, %) = 100 × (g liver weight)/(g body weight); VSI (viscerosomatic index, %) = 100 × (g viscera weight)/(g body weight); CF (condition factor, g/cm^3^) = 100 × (g body weight)/(cm body length)^3^.

**Table 4:** Whole-body proximate composition (wet weigh basis) of GIFT juveniles fed diets with different protein levels under low or high ammonia exposure for 8 weeks.

Ammonia (mg/L)	Protein level (%)	Moisture (%)	Crude protein (%)	Crude lipid (%)	Ash (%)
0.02 mg/L	31.04	65.99 ± 1.02^a^	14.72 ± 0.34^ab^	9.81 ± 0.31^c^	4.08 ± 0.12^cd^
0.88 mg/L	22.64	66.85 ± 0.42^a^	14.50 ± 0.38^a^	9.63 ± 0.83^c^	3.70 ± 0.14^a^
	27.26	67.45 ± 1.10^ab^	14.77 ± 0.33^ab^	9.20 ± 0.42^bc^	3.86 ± 0.21^ab^
	31.04	68.15 ± 1.02^ab^	15.23 ± 0.05^abc^	8.48 ± 0.89^abc^	3.91 ± 0.33^ab^
	35.63	68.72 ± 0.48^abc^	15.25 ± 0.13^abc^	7.71 ± 0.50^ab^	3.86 ± 0.02^bc^
	38.47	69.34 ± 0.77^bc^	15.66 ± 0.65^bc^	6.94 ± 0.52^a^	3.74 ± 0.19^a^
	42.66	69.78 ± 1.07^c^	15.85 ± 0.22^c^	7.09 ± 0.45^a^	3.79 ± 0.29^a^

^∗^Values are mean ± SD (standard deviation) of three replicate per treatment, values in the same row within the same protein level sharing different letters are significantly different at *P* < 0.05, as determined by Tukey's test.

**Table 5:** Partial hematological and serum biochemical indices of GIFT juveniles fed diets with different protein levels under low or high ammonia exposure for 8 weeks.

Ammonia (mg/L)	Protein level (%)	RBC (×10^6^ cells/mm^3^)	Hematocrit (%)	LD (U/L)	AST (U/L)	ALT (U/L)	TP (g/L)
0.02 mg/L	31.04	2.04 ± 0.15^bc^	42.83 ± 4.00^bc^	1448.00 ± 26.87^e^	268.00 ± 14.14^d^	111.50 ± 5.02^c^	36.00 ± 1.37^b^
0.88 mg/L	22.64	1.46 ± 0.13^a^	33.33 ± 1.03^a^	639.33 ± 24.94^a^	118.00 ± 4.24^a^	59.67 ± 3.51^a^	33.00 ± 1.00^a^
	27.26	1.65 ± 0.08^ab^	35.45 ± 0.75^ab^	716.00 ± 36.77^ab^	121.00 ± 4.24^a^	62.00 ± 4.36^a^	33.33 ± 1.15^a^
	31.04	1.47 ± 0.04^a^	36.89 ± 0.85^ab^	893.00 ± 15.55^bc^	191.67 ± 8.62^b^	84.50 ± 4.95^b^	35.33 ± 0.57^ab^
	35.63	1.81 ± 0.03^b^	37.99 ± 1.81^b^	1012.50 ± 84.15^cd^	193.33 ± 3.05^b^	93.33 ± 7.63^bc^	37.33 ± 0.57^b^
	38.47	1.89 ± 0.04^b^	38.20 ± 1.92^b^	1004.00 ± 18.38^cd^	235.00 ± 7.00^c^	105.00 ± 2.00^c^	33.00 ± 1.41^a^
	42.66	1.84 ± 0.01^b^	38.23 ± 0.05^b^	1155.67 ± 92.81^d^	234.33 ± 5.50^c^	107.00 ± 5.65^c^	32.33 ± 1.15^a^

^∗^Values are mean ± SD (standard deviation) of three replicate per treatment, values in the same row within the same protein level sharing different letters are significantly different at *P* < 0.05, as determined by Tukey's test. RBC: red blood cell; LD: lactate dehydrogenase; AST: aspartate transaminase; ALT: alkaline phosphatase; TP: total protein.

**Table 6:** Enzyme activities in liver and gill tissues of GIFT juveniles fed diets with different protein levels under low or high ammonia exposure for 8 weeks.

Ammonia (mg/L)	Protein level (%)	Liver			Gill
		SOD (U/mg)	CAT (U/mL)	GPx (U/mL)	Na^+^/K^+^-ATP (U/mg)
0.02 mg/L	31.04	45.35 ± 5.33^a^	154.19 ± 14.36^d^	35.07 ± 6.25^de^	0.70 ± 0.01^b^
0.88 mg/L	22.64	47.12 ± 0.53^a^	66.78 ± 3.24^a^	22.01 ± 1.45^a^	0.57 ± 0.03^a^
	27.26	48.39 ± 1.23^a^	68.70 ± 0.54^a^	24.67 ± 1.20^ab^	0.56 ± 0.02^a^
	31.04	51.31 ± 2.95^ab^	78.36 ± 2.53^ab^	26.60 ± 1.71^bc^	0.72 ± 0.02^b^
	35.63	54.16 ± 0.69^ab^	97.26 ± 3.03^bc^	30.36 ± 1.34^cd^	0.74 ± 0.03^b^
	38.47	54.90 ± 2.77^ab^	110.79 ± 5.22^c^	32.31 ± 1.21^d^	0.71 ± 0.06^b^
	42.66	61.18 ± 4.59^b^	112.49 ± 7.62^c^	37.47 ± 1.20^e^	0.75 ± 0.02^b^

^∗^Values are mean ± SD (standard deviation) of three replicate per treatment, values in the same row within the same protein level sharing different letters are significantly different at *P* < 0.05, as determined by Tukey's test. SOD: superoxide dismutase; CAT: catalase; GPx: glutathione peroxidase.

**Table 7:** Microstructure of gill and kidney tissues of GIFT juveniles fed diets with different protein levels under low or high ammonia exposure for 8 weeks.

Ammonia (mg/L)	Protein level (%)	Gill			Kidney
		Breadth of gill lamella (*μ*m)	Spacing of gill lamella (*μ*m)	Diameter of chloride cells (*μ*m)	Glomerular long diameter (*μ*m)
0.02 mg/L	31.04	36.12 ± 3.40^ab^	34.08 ± 2.92^ab^	2.27 ± 0.33^ab^	52.08 ± 6.17^ab^
0.88 mg/L	22.64	32.81 ± 0.92^a^	30.98 ± 1.02^a^	1.90 ± 0.21^a^	47.20 ± 1.69^a^
	27.26	33.58 ± 1.28^a^	30.98 ± 1.02^a^	2.00 ± 0.30^a^	47.26 ± 0.43^a^
	31.04	33.71 ± 1.48^a^	35.29 ± 3.14^b^	2.48 ± 0.19^ab^	52.92 ± 1.30^ab^
	35.63	38.62 ± 0.21^ab^	30.86 ± 0.50^a^	2.53 ± 0.12^ab^	53.96 ± 0.95^ab^
	38.47	38.57 ± 2.04^ab^	34.56 ± 0.57^ab^	2.77 ± 0.32^ab^	56.39 ± 0.81^b^
	42.66	44.61 ± 4.97^b^	37.38 ± 1.23^b^	2.55 ± 0.31^b^	64.31 ± 5.58^c^

^∗^Values are mean ± SD (standard deviation) of three replicate per treatment, values in the same row within the same protein level sharing different letters are significantly different at *P* < 0.05, as determined by Tukey's test.

## Data Availability

Data were available from the corresponding authors by reasonable request.
